# The role of various collagen types in tumor biology: a review

**DOI:** 10.3389/fonc.2025.1549797

**Published:** 2025-03-05

**Authors:** Yuchuan Zhou, Zhonghui Jiang, Lu Cao, Jianquan Yang

**Affiliations:** ^1^ Department of Oncology, Affiliated Hospital of North Sichuan Medical College, Nanchong, China; ^2^ Thoracic Surgery, Affiliated Hospital of North Sichuan Medical College, Nanchong, China

**Keywords:** collagen, cancer, review, collagen 1, therapeutic target

## Abstract

Collagen comprises approximately 30% of the body’s protein content and is essential for maintaining the structural integrity, support, and strength of the skin, muscles, bones, and connective tissues. Recent research has further elucidated its role in various aspects of tumor biology, including tumorigenesis, invasion, migration, drug resistance, and recurrence. Furthermore, collagen is involved in prognostic assessments, the evaluation of therapeutic efficacy, immunoregulation, and the identification of potential treatment targets in oncology. This review examines a range of tumor types, including lung, gastric, breast, melanoma, and colorectal cancers, among others. Our objective is to differentiate these tumors based on the specific types of collagen present and to analyze the roles of various collagen types in tumor development, progression, prognosis, and their potential as therapeutic targets.

## 1 Introduction

Collagen, a fundamental component of the extracellular matrix (ECM), is ubiquitously distributed across various organs and tissues, playing a crucial role in maintaining the structural integrity of the ECM (1, 2). To date, 28 distinct collagen types have been identified, each differing in molecular structure, associated cellular components, and specific anatomical distribution (3). Collagen is predominantly secreted by fibroblasts, myoblasts, adipocytes, endothelial cells, and certain epithelial cells, and is vital for wound healing and tissue repair due to its capacity to facilitate cell migration and proliferation (4–7). Furthermore, collagen acts as a signaling molecule, regulating a wide array of cellular processes, including differentiation, modulation of inflammatory responses, immune function, and gene expression, thereby contributing to the maintenance of overall physiological homeostasis (8–15).

Clinical evidence indicates that chemotherapy and immunotherapies exhibit significantly diminished efficacy in treating solid tumors compared to hematological malignancies (16, 17). These findings highlight the distinct role of the tumor microenvironment (TME) in reducing therapeutic effectiveness in solid tumors. Collagen is a pivotal component of the TME. A diverse array of cell types, including cancer cells, cancer-associated fibroblasts (CAFs), endothelial cells, and immune cells, contributes substantially to collagen synthesis (18). These cells produce procollagen, which is subsequently secreted into the ECM and processed by proteases into mature collagen fibers (19). Collagen is the primary determinant of the density and stiffness of the TME and has been implicated in tumorigenesis, progression, invasion, metastasis, drug resistance, and immunoregulation (20–24). Additionally, alterations in collagen organization within the tumor microenvironment have been associated with prognostic outcomes. Changes in collagen characteristics—such as content, maturity, morphology, and architecture—have been correlated with prognosis, indicating that collagen width may serve as a significant prognostic marker (25–29).

Strategies targeting collagen depletion have been proposed to enhance drug delivery in solid tumors. In particular, collagen depletion has been explored as a strategy to improve drug delivery and increase the effectiveness of phototherapy in pancreatic cancer (30–32). Moreover, its potential impact on modulating the efficacy of immunotherapy has been relatively understudied. Recent advancements in the investigation of the ECM have highlighted the role of collagen in promoting an immunosuppressive TME and serving as a physical barrier that protects tumors from immunotherapy (33, 34). Additionally, research has shown that tumor-associated macrophages contribute to the progression of bladder tumors by secreting Type I collagen, which subsequently activates the pro-survival PI3K/AKT signaling pathway (35). Furthermore, changes in collagen have been examined in relation to postoperative anastomotic stenosis following neoadjuvant chemoradiotherapy for rectal cancer.

This review aims to deliver an exhaustive analysis of the recent roles of various collagen types across different tumor classifications, thereby establishing a foundation for personalized and precise tumor therapies.

## 2 The role of collagen types in tumors and current treatment methods

### 2.1 Type I collagen

COL1 is the most prevalent member of the collagen family, predominantly located in tissues such as skin, bones, muscles, and blood vessels. It plays a critical role in wound healing and tissue regeneration. In the context of cancer, COL1 is often associated with tumor progression and aggressiveness, COL1 mainly secreted by CAF and significantly contributes to the TME, It not only impedes the infiltration of tumor-immune cells and therapeutic agents via mechanical forces but also enhances tumor growth through intermolecular interactions across various tumor types (36–41). COL1 supports glioblastoma stem cells (GSCs) by enhancing CD133-p85 interaction and Akt phosphorylation. This boosts ATF4 protein levels, increasing SLC1A5-dependent glutamine uptake and glutathione synthesis, thereby promoting GSC self-renewal and tumor growth (42). On the contrary, research indicates that COL1-mediated tumor suppression counteracts the tumor-promoting effects ofCAF, underscoring the complex interactions within the tumor stroma (43, 44). COL1 also possesses prognostic value in cancers such as gastric, colorectal, and breast cancer (45, 46), particularly serving as an independent predictor of 5-year overall survival and recurrence-free survival in patients with TNM stage II/III colorectal cancer undergoing surgical treatment (47).

The abundant presence of COL1 facilitates its deposition and cross-linking, resulting in the formation of a rigid and dense physical barrier. This barrier restricts drug penetration and immune cell infiltration, thereby contributing to drug resistance and metabolic adaptations. Consequently, COL1 has been identified as a therapeutic target in cancer treatment, with approaches such as collagenase treatment, protein-free collagen degradation, collagen-degrading bacteria, and the degradation of the tumor extracellular matrix mediated by armed oncolytic viruses, all demonstrating significant efficacy (48–51). Furthermore, the status of collagen crosslinking is a significant determinant of tissue stiffness. This can be influenced by modulating the enzymatic activity of lysyl oxidase and altering collagen glycation-related crosslinking (52, 53). Additionally, there is growing interest in repurposing approved antifibrotic medications, which target collagen synthesis and maturation, as anticancer drugs. For example, halofuginone, an antifibrotic agent, inhibits the activation of Smad3 in fibroblasts in response to transforming growth factor beta (TGF-β1) stimulation. COL1 contains collagen-binding domains (CBD), which can be utilized in the engineering of antibodies, drugs, or cytokines. Incorporating a CBD into these therapeutic agents facilitates their targeted delivery and release within the tumor collagen scaffold. This approach minimizes off-target effects, reduces systemic toxicity, and enhances localized retention, thereby improving therapeutic efficacy. Additionally, COL1 is implicated in various facets of cancer biology. For instance, the mineralization of COL1 influences the glycocalyx of breast cancer cells, potentially affecting NK cell-mediated cytotoxicity (54). Studies have identified a homotrimer COL1 signature linked to clinical manifestations and tertiary lymphoid structures in liver cancer (55). Furthermore, a COL1-specific imaging probe has been utilized to assess treatment response in pancreatic cancer, underscoring the role of tumor-associated fibrosis in disease progression (56). Moreover, COL1 has been associated with apoptotic balance in hepatocellular carcinoma cells, highlighting the complex interplay between collagen types and apoptotic responses in cancer (57). Modulating STAT3 signaling in COL1-positive fibroblasts presents a promising strategy for managing colitis-associated cancer, highlighting the therapeutic potential of targeting collagen-expressing fibroblasts in oncological treatments. Collectively, these studies emphasize the critical role of COL1 in tumor biology and its interactions with stroma cells, offering valuable insights into potential diagnostic, prognostic, and therapeutic applications across various cancer types.

### 2.2 Type III collagen

COL3 is the second most prevalent collagen in tissues and is frequently co-expressed with COL1, also secreted by CAF. Notably, COL3 primarily functions as a tumor suppressor and serves as a prognostic marker. Previous studies have suggested that a collagen-rich peritumoral stroma, particularly rich in COL3, may adversely affect the interaction between T cells and tumor cells. This interaction can lead to immune exclusion and potential resistance mechanisms in patients with metastatic melanoma following PD-1 inhibition (58). Furthermore, COL3 has been demonstrated to exert a tumor-restrictive effect in human breast cancer. In biopsy samples of human triple-negative breast cancer, a study reveals an increased deposition of COL3 compared to COL1 in non-invasive regions as opposed to invasive regions. Correspondingly, a bioinformatics analysis of over 1,000 breast cancer patient biopsies from The Cancer Genome Atlas BRCA cohort indicates that patients exhibiting higher COL3:COL1 expression ratios in bulk tumor samples experience enhanced overall, disease-free, and progression-free survival compared to those with elevated COL1:COL3 expression ratios (59). In a similar vein, recombinant humanized COL3 has been shown to inhibit ovarian cancer and induce protective anti-tumor immunity by modulating autophagy through GSTP1 (60).

Furthermore, dormant cancer cells establish an ECM niche enriched with COL3, which interacts with discoidin domain receptor 1 (DDR1) to induce and maintain cellular dormancy (61). The differential expression of COL3A1 in human cancer tissues compared to normal samples underscores its positive correlation with tumor-infiltrating immune cells (62). A recent review has discussed the prognostic value of quantifying tumor fibrosis by measuring the pro-peptide of COL3 in serum across various solid tumor types, highlighting its significance in understanding prognosis and guiding future drug development efforts (63). An enzyme-linked immunosorbent assay (ELISA) targeting a circulating COL3 fragment, derived from fibroblast activation protein (FAP) cleavage, has been developed to reflect FAP activity in patients with non-small cell lung cancer and spondyloarthritis (64). Additionally, keloid-like collagen, which contains both COL3 and COL1, has been evaluated for its diagnostic and prognostic significance in the remodeling patterns of the extracellular matrix in colorectal cancer, emphasizing the importance of quantitative visual assessment in determining prognosis (47). Furthermore, digital whole slide image analysis was employed using COL3 to assess the prognostic significance of stromal markers in triple-negative breast cancer (46). In conclusion, COL3 seems to have a substantial impact on tumor biology, influencing diagnosis, prognosis, and therapeutic approaches across different cancer types. Further investigation into the interactions between COL3 and tumors is necessary to enhance our understanding of its potential as a target for cancer treatment.

### 2.3 Type IV collagen

COL4, a key component of the basement membrane (BM), is critically involved in tumor progression and therapeutic response. COL4 has been shown to facilitate malignant cell proliferation. Three-dimensional (3D) biomimetic cell culture platforms utilizing COL4 pre-treated hydrogels have been found to support the proliferation of glioblastoma cells (65). One study assessed COL4 levels in patients with clear cell renal cell carcinoma (ccRCC) and proposed that variations in COL4 concentrations may contribute to malignant tumor progression (66). Another study explored the association between COL4 and hepatocellular carcinoma (HCC) prognosis in patients undergoing hepatectomy, indicating a potential link between COL4 levels and HCC malignant behaviors (67). Conversely, it has been demonstrated that activated T-cells release granzyme B (GzB), which can cleave type IV collagen during tumor infiltration. This finding suggests that measuring GzB-generated type IV collagen degradation fragments in liquid biopsies could be useful for identifying metastatic melanoma patients who are responding to immune checkpoint inhibitor therapy (58). Furthermore, the α4 NC1 domain of type IV collagen, referred to as Tetrastatin, has been demonstrated to inhibit tumor growth in a mouse melanoma model by attenuating VEGF-induced angiogenesis (68). In the context of breast cancer, type IV collagen has been associated with tumor progression and the regulation of metabolism. The α5 chain of type IV collagen is predominantly expressed in luminal-type breast cancer and is regulated by estrogen receptor-α, functioning as a microenvironmental regulator that influences cancer cell metabolism (69). Additionally, stromal expression of COL4 has been recognized as a prognostic indicator in small invasive breast cancers (70). Collectively, COL4 and its degradation products are integral to tumor biology, immune response, and therapeutic outcomes across various cancer types, including melanoma and breast cancer. Elucidating the interactions between COL4 and the TME may offer valuable insights for the development of innovative therapeutic strategies and biomarkers for cancer diagnosis and treatment monitoring.

### 2.4 Type VI collagen

COL6 establishes a filamentous network within connective tissue, facilitating the linkage between matrix macromolecules and cells. It interacts with COL5 and fibronectin, thereby enhancing the structural integrity of tissue scaffolds. Additionally, COL6 plays a crucial role in mediating cell adhesion and promoting cellular migration. COL6 has been implicated in various aspects of tumor progression and metastasis. In breast cancer research, it has been discovered that oncogenic linear COL6 in invasive breast cancer is induced by CCL5, suggesting a potential role in the disease’s progression (71). Similarly, it has been demonstrated that COL6 regulates the CDK4/6-p-Rb signaling pathway, thereby promoting ovarian cancer invasiveness, stemness, and metastasis (72). Additionally, COL6 deposition primes the glioblastoma microenvironment for invasion through mechanostimulation of β-catenin signaling. Conversely, a study indicated that COL6-α1 and COL6-α2 suppress the proliferation, migration, and invasion of bladder cancer cells, highlighting the complex role of collagen VI in different cancer types (73).

Through the application of machine learning techniques, researchers identified epithelial-mesenchymal transition (EMT)-related genes associated with cervical cancer etiology and prognosis, with COL6 emerging as a potential prognostic indicator in cervical cancer (74). Additionally, a study constructed a reliable five-gene prognostic signature for colon adenocarcinoma associated with the wild-type allelic state of the COL6A6 gene (75). By identifying independent prognostic signatures based on allelic variations, the researchers elucidated significant prognostic disparities between high-risk and low-risk patient cohorts. This finding implies that the genetic context associated with collagen genes may influence cancer prognosis. Furthermore, a study demonstrated that sarcoma cells secrete hypoxia-modified COL6, which compromises the integrity of the lung endothelial barrier and facilitates metastasis, thereby indicating a direct involvement of COL6 in promoting cancer cell migration and invasion (76). Additionally, research has shown that immune cells contribute to the dynamics of mammary collagen during pregnancy, suggesting a potential connection between collagen regulation and immune responses within the tumor microenvironment (77). In conclusion, investigations into COL6 and its interactions with tumors underscore.

### 2.5 Type X collagen

COL10 is a relatively understudied collagen type in the context of cancer, particularly when compared to other collagen types such as types I, III, and V. Nonetheless, recent research has begun to elucidate the potential role of COL10 in cancer progression. The COL10 α1 chain (COL10A1), which encodes the α−1 chain of COL10, plays a crucial role in providing tensile strength and structural integrity to tissues. The upregulation of COL10A1 has been shown to facilitate the progression of triple-negative breast cancer through the Wnt/β-catenin signaling pathway (78). In colorectal, prostate, pancreatic, and gastric cancers, COL10A1 is associated with poor prognosis and is linked to tumor vasculature (79–85). Furthermore, COL10 has been found to facilitate prostate cancer progression by interacting with INHBA to activate the PI3K/AKT pathway (81). Additionally, stromal COL10A1 and tumor-infiltrating lymphocytes have been identified as potential predictive markers for neoadjuvant therapy in estrogen receptor-positive/HER2-positive breast cancer (86, 87).The COL10A1 gene facilitates the stratification of colon cancer invasiveness and is associated with the enrichment of the extracellular matrix and immune cells within the tumor parenchyma (88). Furthermore, the secretion of COL10A1 in lung cancer cells promotes metastasis and attracts cancer-associated fibroblasts (89). Additionally, miR-384 induces apoptosis and autophagy in non-small cell lung cancer cells by negatively regulating the COL10A1 gene (90).

### 2.6 Type XI collagen

COL11 has been implicated in various tumor types, particularly osteochondrogenic tumors (91). The expression of COL2 and COL11 has been observed in canine mammary mixed tumors, where tumor cells exhibited collagen production in collagen gel culture (92). A related study demonstrated that COL11A1-regulated hypermethylation leads to the downregulation of miR-509-3p expression, thereby facilitating cancer progression and chemoresistance in ovarian cancer cells. This research elucidates the complex mechanisms involving COL11A1 in cancer progression (93). Furthermore, COL11 has been identified as a potential biomarker in cancer research, with noninvasive biomarkers targeting the propeptides of the COL11 alpha-1 chain (PRO-C11) being developed for patients with pancreatic ductal adenocarcinoma, enabling the quantification of COL11 production (94, 95). Additionally, COL11 was found to be significantly upregulated in lung adenocarcinoma tissues compared to non-tumor tissues, suggesting its potential involvement in this type of cancer (96). Furthermore, the interaction between COL11 and tumor cells has been investigated within the framework of lung cancer. COL11 has been shown to inhibit the functions of lung cancer-associated fibroblasts and limit the availability of integrin binding sites on the COL1 matrix, suggesting its potential role in modulating the tumor microenvironment (97). The existing literature indicates that COL11 may play a crucial role in tumorigenesis and could serve as a valuable biomarker for cancer diagnosis and prognosis. Additional research is required to elucidate the specific mechanisms by which COL11 is involved in various tumor types.

### 2.7 Type XVII collagen

COL17, also referred to as BP180, along with integrin α6β4, CD151, plectin, and BP230, constitutes essential components of type I hemidesmosomes (HDs). These components play a pivotal role in mediating interstitial adhesion, facilitating cell polarization, and maintaining the spatial organization of tissue architecture. Furthermore, COL17 is significantly implicated in various aspects of tumor biology. Notably, it has been shown to regulate tumor growth in pancreatic and colorectal cancers (20). Specifically, COL17 promotes the dormancy of colorectal cancer cells by activating mTORC2 signaling (98). Conversely, it has been reported that COL17 inhibits the proliferation and growth of breast cancer cells through the deactivation of the AKT/mTOR signaling pathway (99). Additionally, a study indicated that elevated levels of the ectodomain of COL17 in circulation serve as a prognostic marker for patients with metastatic colorectal cancer, suggesting that PRO-C17 could be utilized as a non-invasive biomarker to elucidate tumor heterogeneity and its role in tumor progression (100). Similarly, type COL17 collagen may serve as a tumor biomarker in head and neck squamous cell carcinoma. A study has highlighted the tissue-specific expression of type COL17 isoforms and their molecular interactions with P120 catenin, underscoring their role in epidermal keratinocyte cell-cell adhesion (101). Furthermore, COL17 has been identified as a potential target for fluorescence-guided surgery in non-small cell lung cancers, highlighting its significance as a cell surface biomarker in tumor tissues (102). Collectively, these studies emphasize the critical role of type COL17 collagen in tumor biology and its potential as a prognostic marker across various cancer types.

### 2.8 Other types of collagen associated with cancer

The literature on COL5, COL7, COL9, and COL12 in the context of tumors is limited, with only a few studies addressing their functions and roles as tumor markers. Consequently, numerous functions and underlying mechanisms remain poorly understood.

Type V collagen (COL5) is a regulatory fibril-forming collagen that plays a relatively minor role within the ECM. COL5 is synthesized in various mesenchymal cells and its gene expression is modulated by TGF-β and growth factors (105). Morphologically, COL5 is incorporated into heterotypic fibrils in conjunction with COL1. In the context of oncogenesis, myofibroblast-derived COL5 contributes to elevated mechanical stress within tissues, thereby promoting metastasis and resistance to therapy in lung adenocarcinoma cells (106). Additionally, COL5A1 has been implicated in the progression of triple-negative breast cancer through the activation of tumor cell-macrophage crosstalk (107). Furthermore, increased expression of COL5A2, a gene encoding a type V collagen alpha chain, has been correlated with poor survival in gastric cancer (109) and colorectal cancer by upregulation of Wnt/β-catenin signaling (108). As a prognostic biomarker, COL5 holds potential for predicting outcomes in pancreatic ductal adenocarcinoma, gastric cancer, laryngeal squamous cell carcinoma, and cervical cancer (110–112).

Type VII collagen (COL7) constitutes a principal component of anchoring fibrils, situated beneath the basal lamina within the dermal-epidermal basement membrane zone of the skin. The diagnostic potential of COL7A1 has also been explored in oral squamous cell carcinoma (OSCC) and oral leukoplakia (OL). Poomsawat et al. conducted a comprehensive study involving 254 samples, revealing that COL7A1 expression was significantly reduced in OSCC compared to normal oral mucosa and OL without dysplasia (113). The research conducted by Oh et al. has demonstrated a significant correlation between COL7A1 expression and the prognosis of patients with gastric cancer. Utilizing DNA microarray analysis, the study compared gene expression profiles between cancerous and non-cancerous gastric tissues. The findings revealed a marked upregulation of COL7A1 in gastric cancer tissues relative to normal tissues. Moreover, patients with intracellular COL7A1 expression exhibited a substantially lower five-year overall survival (OS) rate of 41.5%, in contrast to 69.7% for those with solely extracellular expression. This indicates that the localization of COL7A1 expression serves as an independent prognostic factor, with a hazard ratio of 2.00 (95% CI 1.26-3.16, p=0.003) for OS (114).

Type IX collagen (COL9) is a constituent of the low-abundance collagen family, specifically categorized as FACITs, predominantly localized in cartilage. Its function within the tumor microenvironment remains largely unexamined. To explore the potential of COL9 as a biomarker in oncology, an immunoassay named PRO-C9 was developed, and its technical performance was evaluated for serum analysis. PRO-C9 concentrations were quantified in serum samples from 259 patients diagnosed with various solid tumor types and compared to serum levels from 73 healthy controls. The results indicated that PRO-C9 levels were significantly elevated in patients with solid tumors, including those of the bladder, breast, colorectal, gastric, head and neck, lung, melanoma, ovarian, pancreatic, and renal origins, in comparison to levels observed in healthy controls (115).

Type XII collagen (COL12) has emerged as a potential biomarker for solid tumors due to its elevated levels in the serum of affected patients. A study has developed an ELISA to quantify circulating COL12, underscoring its potential as a non-invasive tool for detecting activated fibroblasts within the tumor microenvironment (103). Moreover, the integrated analysis of mass spectrometry and single-cell transcriptomics reveals that COL12, secreted by CAFs, alters the organization of COL1. This modification contributes to the formation of a pro-invasive microenvironment that promotes metastatic dissemination in breast cancer. Therefore, COL12 has the potential to serve as a biomarker for identifying breast cancer patients who are at an increased risk of metastatic relapse (104).

## 3 Conclusions and future perspectives

**Figure 1:**
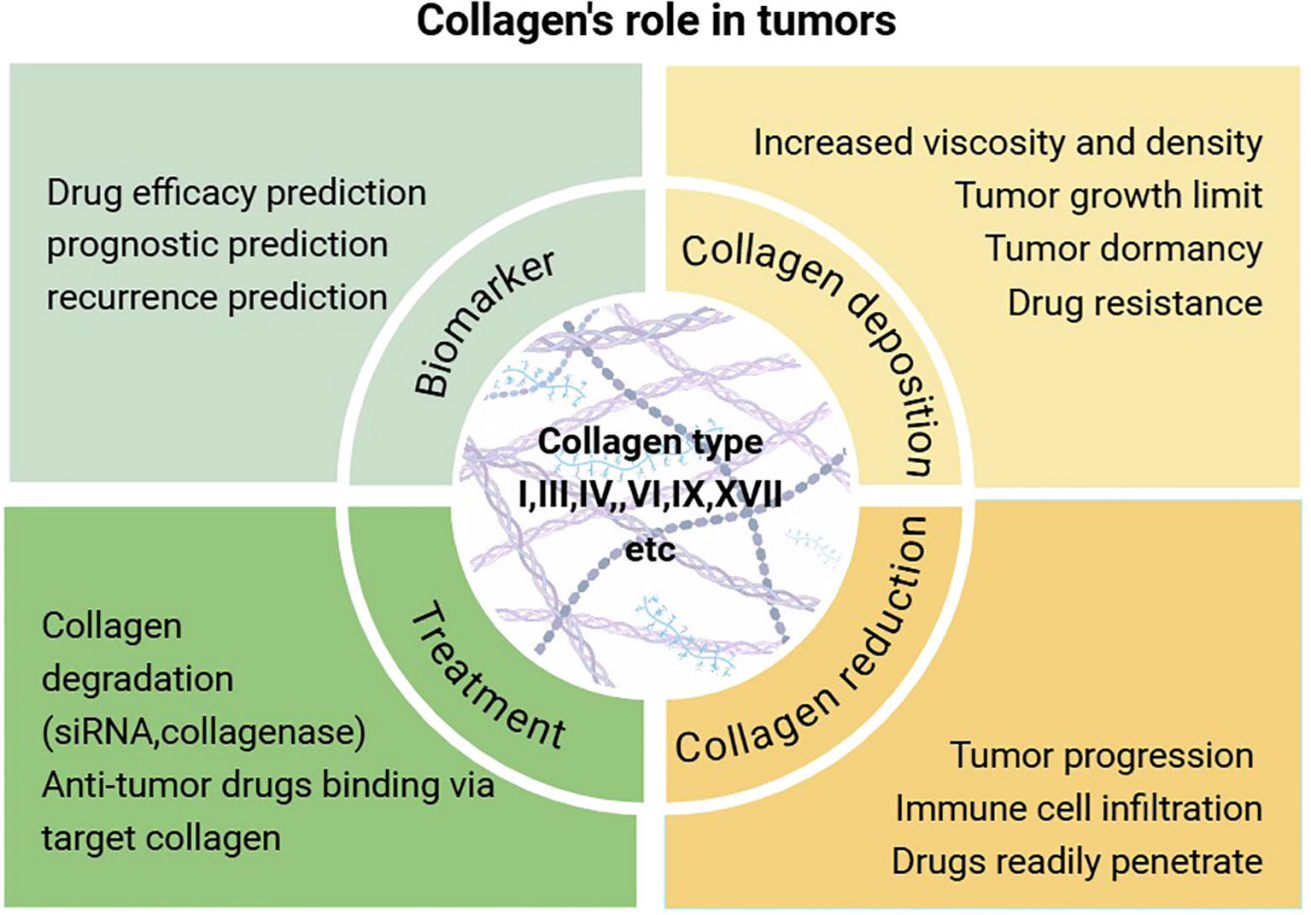
An examination of the biological role of collagen in tumorigenesis and an overview of contemporary therapeutic approaches.

In addition to serving as a structural scaffold for tissues and cells, collagen plays a significant role in various biophysical, biochemical, and cellular alterations that profoundly influence cancer behavior. The precise mechanisms by which collagen operates within tumors remain inadequately understood, with different collagen types exhibiting distinct functions across various tumor types (**Table 1**). Broadly, the mechanisms of collagen’s action in tumors can be categorized as follows: (1) promotion of tumor growth, invasion, and metastasis; (2) enhancement of extracellular matrix viscosity, which inhibits tumor invasion and metastasis but results in diminished efficacy of chemotherapy and immunotherapy; (3) monitoring of tumor recurrence, serving as a marker for distinguishing between benign and malignant tumors and for prognostication; and (4) targeting in cancer therapy, either through direct collagen degradation or as a target for anti-tumor drugs *in vivo* (**Figure 1**).

**Table 1:** Collagens in tumor biology.

Types	Mechanism	Tumor progression/suppressor	Source	Clinical application
COL1	Mechanical limitation and immunity regulation	Both (Primarily promotes tumors)	Secreted by CAFs	Antitumor target/biomarkers
COL3	Unclear	Tumor suppressor	Secreted by CAFs	Biomarkers
COL4	Unclear	Tumor progression	Component of basement membrane	Biomarkers
COL5	Regulatory fibril-forming	Tumor progression	Secreted by mesenchymal cells	Biomarkers
COL6	Unclear	Both	Located between the basement membrane and interstitial matrix	Biomarkers
COL7	Unclear	Unclear	Forming fibrils that anchor the epidermis to the dermis	Biomarkers
COL9	Unclear	Unclear	Unclear	Biomarkers
COL10	TME stiffness and structural integrity to tissues	Tumor progression	Bones and hypertrophic cartilage	Biomarkers
COL11	Unclear	Tumor progression	Unclear	Biomarkers
COL12	Unclear	Tumor progression	Secreted by CAF (104)	Biomarkers
COL17	Regulate tumor proliferation, metastasis and dormancy	Both	Components of type I hemidesmosomes	Aiding cancer therapy/biomarkers

An overview of the mechanisms, functions, sources, and clinical applications of various collagen types in tumor biology.

Detecting collagen content in primary sites and body fluids using traditional methods is relatively straightforward. However, leveraging collagen detection results to predict tumor recurrence, metastasis, and evaluate treatment efficacy requires further in-depth investigation. This is particularly important given that the type, spatial distribution, and geometric characteristics of collagen significantly influence the biological behavior of tumors. Recent advancements in visualization techniques, such as second-harmonic generation imaging and optical coherence tomography (60, 116, 117), enable detailed characterization of collagen without the need for staining, offering promising potential for clinical application. Furthermore, emerging technologies, including single-cell sequencing, mass spectrometry, and CRISPR-Cas9, have demonstrated significant advantages in the investigation of cancer-associated fibroblasts (CAFs), collagen, and tumor biology. This is particularly evident in pancreatic and breast tumors, which are characterized by a high collagen content (118).

Current collagen-targeted therapies primarily focus on reducing collagen expression and lack strategies to modulate or “normalize” the spatial distribution and geometric characteristics of collagen within the TME. Furthermore, current collagen-targeted therapies may impact non-tumor tissues, potentially resulting in significant side effects. Nevertheless, ongoing advancements and interdisciplinary collaborations in cell biology, oncology, and nanotechnology are likely to facilitate the translation of additional collagen-targeting strategies into clinical applications.

## References

[1] Guillard J Schwörer S. Metabolic control of collagen synthesis. Matrix Biol. (2024) 133:43–56. doi: 10.1016/j.matbio.2024.07.003 39084474 PMC11402592

[2] Fu Y Zhou Y Wang K Li Z Kong W. Extracellular matrix interactome in modulating vascular homeostasis and remodeling. Circ Res. (2024) 134(7):931–49. doi: 10.1161/CIRCRESAHA.123.324055 38547250

[3] Sorushanova A Delgado LM Wu Z Shologu N Kshirsagar A Raghunath R. The collagen suprafamily: From biosynthesis to advanced biomaterial development. Adv Mater. (2019) 31(1):e1801651. doi: 10.1002/adma.201801651 30126066

[4] Deshmukh SN Dive AM Moharil R Munde P. Enigmatic insight into collagen. J Oral Maxillofac Pathol. (2016) 20(2):276–83. doi: 10.4103/0973-029X.185932 PMC4989561 27601823

[5] Franchi M Piperigkou Z Mastronikolis NS Karamanos N. Extracellular matrix biomechanical roles and adaptation in health and disease. FEBS J. (2024) 291(3):430–40. doi: 10.1111/febs.16938 37612040

[6] Lee JH Won YJ Kim H Choi M Lee E Ryoou B. Adipose tissue-derived mesenchymal stem cell-derived exosomes promote wound healing and tissue regeneration. Int J Mol Sci. (2023) 24(13). doi: 10.3390/ijms241310434 PMC10341478 37445612

[7] Brisson BK Stewart DC Burgwin C Chenoweth D Wells RG Adams SL. Cysteine-rich domain of type III collagen n-propeptide inhibits fibroblast activation by attenuating TGFβ signaling. Matrix Biol. (2022) 109:19–33. doi: 10.1016/j.matbio.2022.03.004 35339637 PMC9086147

[8] Dev A Jr. Vachher M and Prasad CP. β-catenin inhibitors in cancer therapeutics: intricacies and way forward. Bioengineered. (2023) 14(1):2251696. doi: 10.1080/21655979.2023.2251696 37655825 PMC10478749

[9] Aggarwal A Khalighi S Babu D Li H Azarianpour-Esfahani S Corredor G. Computational pathology identifies immune-mediated collagen disruption to predict clinical outcomes in gynecologic malignancies. Commun Med (Lond). (2024) 4(1):2. doi: 10.1038/s43856-023-00428-0 38172536 PMC10764846

[10] Kang R Tang D. PKR-dependent inflammatory signals. Sci Signal. (2012) 5(247):pe47. doi: 10.1126/scisignal.2003511 23092889 PMC3656404

[11] Sapudom J Alatoom A Tipay PS Teo JC. Matrix stiffening from collagen fibril density and alignment modulates YAP-mediated T-cell immune suppression. Biomaterials. (2025) 315:122900. doi: 10.1016/j.biomaterials.2024.122900 39461060

[12] Marzban S Srivastava S Kartika S Bravo R Safriel R Zarski A. Spatial interactions modulate tumor growth and immune infiltration. NPJ Syst Biol Appl. (2024) 10(1):106. doi: 10.1038/s41540-024-00438-1 39349537 PMC11442770

[13] Ochs MJ Steinhilber D and Suess B. MicroRNA involved in inflammation: Control of eicosanoid pathway. Front Pharmacol. (2011) 2:39. doi: 10.3389/fphar.2011.00039 21811464 PMC3142860

[14] Liu J Chiang HC Xiong W Laurent V Griffiths SC Dülfer J. A highly selective humanized DDR1 mAb reverses immune exclusion by disrupting collagen fiber alignment in breast cancer. J Immunother Cancer. (2023) 11(6). doi: 10.1136/jitc-2023-006720 PMC10277525 37328286

[15] He R He Z Zhang T Liu B Gao M Li N. HDAC3 in action: Expanding roles in inflammation and inflammatory diseases. Cell Prolif. (2025) 58(1):e13731. doi: 10.1111/cpr.13731 39143689 PMC11693555

[16] Zheng R Shen K Liang S Lyu Y Zhang S Dong H. Specific ECM degradation potentiates the antitumor activity of CAR-T cells in solid tumors. Cell Mol Immunol. (2024) 21(12):1491–504. doi: 10.1038/s41423-024-01228-9 PMC11606952 39472748

[17] Zhao Y Shen M Wu L Yang H Yao Y Yang Q. Stromal cells in the tumor microenvironment: accomplices of tumor progression? Cell Death Dis. (2023) 14(9):587. doi: 10.1038/s41419-023-06110-6 37666813 PMC10477351

[18] De Martino D Bravo-Cordero JJ. Collagens in cancer: Structural regulators and guardians of cancer progression. Cancer Res. (2023) 83(9):1386–92. doi: 10.1158/0008-5472.CAN-22-2034 PMC10159947 36638361

[19] Bernabei I Faure E Romani M Wegrzyn J Brinckmann J Chobaz V. Inhibiting lysyl oxidases prevents pathologic cartilage calcification. BioMed Pharmacother. (2024) 171:116075. doi: 10.1016/j.biopha.2023.116075 38183742

[20] Cao JH Cao CH Lin JL Li SY He LJ Han K. NEIL1 drives the initiation of colorectal cancer through transcriptional regulation of COL17A1. Cell Rep. (2024) 43(1):113654. doi: 10.1016/j.celrep.2023.113654 38175757

[21] Liotta LA Abe S Robey PG Martin GR. Preferential digestion of basement membrane collagen by an enzyme derived from a metastatic murine tumor. Proc Natl Acad Sci USA. (1979) 76(5):2268–72. doi: 10.1073/pnas.76.5.2268 PMC383580 221920

[22] Brisson BK Mauldin EA Lei W Vogel LK Power AM Lo A. Type III collagen directs stromal organization and limits metastasis in a murine model of breast cancer. Am J Pathol. (2015) 185(5):1471–86. doi: 10.1016/j.ajpath.2015.01.029 PMC4419211 25795282

[23] Togni L Furlani M Belloni A Riberti N Giuliani A Notarstefano V. Biomolecular alterations temporally anticipate microarchitectural modifications of collagen in oral tongue squamous cell carcinoma. iScience. (2024) 27(7):110303. doi: 10.1016/j.isci.2024.110303 39040062 PMC11261445

[24] Tang L Xu YC Zhang FC. Review of the role of collagen in tumorigenesis and development. J Shanghai Jiao Tong Univ (Medical Science). (2023) 43(12):1577–84. doi: 10.3969/j.issn.1674-8115.2023.12.014

[25] Zhou ZH Ji CD Xiao HL Zhao HB Cui YH Bian XW. Reorganized collagen in the tumor microenvironment of gastric cancer and its association with prognosis. J Cancer. (2017) 8(8):1466–76. doi: 10.7150/jca.18466 PMC5479253 28638462

[26] Hompland T Erikson A Lindgren M Lindmo T de Lange Davies C. Second-harmonic generation in collagen as a potential cancer diagnostic parameter. J BioMed Opt. (2008) 13(5):054050. doi: 10.1117/1.2983664 19021430

[27] Riegler J Labyed Y Rosenzweig S Javinal V Castiglioni A Dominguez CX. Tumor elastography and its association with collagen and the tumor microenvironment. Clin Cancer Res. (2018) 24(18):4455–67. doi: 10.1158/1078-0432.CCR-17-3262 29798909

[28] Li X Shepard HM Cowell JA Zhao C Osgood RJ Rosengren S. Parallel accumulation of tumor hyaluronan, collagen, and other drivers of tumor progression. Clin Cancer Res. (2018) 24(19):4798–807. doi: 10.1158/1078-0432.CCR-17-3284 PMC6743334 30084839

[29] Feng M Wang H Zheng J Chen Z Kang B Zhao Y. Association of collagen changes in distal anastomotic margin and anastomotic stenosis after neoadjuvant chemoradiotherapy for rectal cancer. J Am Coll Surg. (2024) 239(4):363–74. doi: 10.1097/XCS.0000000000001116 38752618

[30] Li D Chen X Dai W Jin Q Wang D Ji J. Photo-triggered cascade therapy: A NIR-II AIE luminogen collaborating with nitric oxide facilitates efficient collagen depletion for boosting pancreatic cancer phototheranostics. Adv Mater. (2024) 36(13):e2306476. doi: 10.1002/adma.202306476 38157423

[31] Yu B Wang W Zhang Y Sun Y Li C Liu Q. Enhancing the tumor penetration of multiarm polymers by collagenase modification. Biomater Sci. (2024) 12(9):2302–11. doi: 10.1039/D3BM02123H 38497169

[32] Kato M Hattori Y Kubo M Maitani Y. Collagenase-1 injection improved tumor distribution and gene expression of cationic lipoplex. Int J Pharm. (2012) 423(2):428–34. doi: 10.1016/j.ijpharm.2011.12.015 22197775

[33] Flies DB Langermann S Jensen C Karsdal MA Willumsen N. Regulation of tumor immunity and immunotherapy by the tumor collagen extracellular matrix. Front Immunol. (2023) 14:1199513. doi: 10.3389/fimmu.2023.1199513 37662958 PMC10470046

[34] Hong Y Lv Z Xing Z Xu H Chand H Wang J. Identification of molecular subtypes and diagnostic model in clear cell renal cell carcinoma based on collagen-related genes may predict the response of immunotherapy. Front Pharmacol. (2024) 15:1325447. doi: 10.3389/fphar.2024.1325447 38375034 PMC10875022

[35] Qiu S Deng L Liao X Nie L Qi F Jin K. Tumor-associated macrophages promote bladder tumor growth through PI3K/AKT signal induced by collagen. Cancer Sci. (2019) 110(7):2110–8. doi: 10.1111/cas.14078 PMC6609800 31120174

[36] Graf F Horn P Ho AD Boutros M Maercker C. The extracellular matrix proteins type I collagen, type III collagen, fibronectin, and laminin 421 stimulate migration of cancer cells. FASEB J. (2021) 35(7):e21692. doi: 10.1096/fj.202002558RR 34118087

[37] Liu J Zhang X Yang M Zhang X. CircCOL1A1 promotes proliferation, migration, and invasion of colorectal cancer (CRC) cells and glutamine metabolism through GLS1 up-regulation by sponging miR-214-3p. J Cancer Res Clin Oncol. (2024) 150(4):211. doi: 10.1007/s00432-024-05736-z 38662258 PMC11045592

[38] Zhong C Tao B Tang F Yang X Peng T You J. Remodeling cancer stemness by collagen/fibronectin *via* the AKT and CDC42 signaling pathway crosstalk in glioma. Theranostics. (2021) 11(4):1991–2005. doi: 10.7150/thno.50613 33408794 PMC7778591

[39] Chen Y Yang S Tavormina J Tampe D Zeisberg M Wang H. Oncogenic collagen I homotrimers from cancer cells bind to α3β1 integrin and impact tumor microbiome and immunity to promote pancreatic cancer. Cancer Cell. (2022) 40(8):818–834.e9. doi: 10.1016/j.ccell.2022.06.011 35868307 PMC9831277

[40] Mizuno S Bustos MA Hayashi Y Abe K Furuhashi S Naeini Y. Induced collagen type-I secretion by hepatocytes of the melanoma liver metastasis is associated with a reduction in tumour-infiltrating lymphocytes. Clin Transl Med. (2024) 14(11):e70067. doi: 10.1002/ctm2.70067 39496484 PMC11534464

[41] Shields MA Dangi-Garimella S Krantz SB Bentrem DJ Munshi HG. Pancreatic cancer cells respond to type I collagen by inducing snail expression to promote membrane type 1 matrix metalloproteinase-dependent collagen invasion. J Biol Chem. (2011) 286(12):10495–504. doi: 10.1074/jbc.M110.195628 PMC3060503 21288898

[42] Wei Y Geng S Si Y Yang Y Chen Q Huang S. The interaction between collagen 1 and high mannose type CD133 up-regulates glutamine transporter SLC1A5 to promote the tumorigenesis of glioblastoma stem cells. Adv Sci (Weinh). (2024) 11(3):e2306715. doi: 10.1002/advs.202306715 37997289 PMC10797482

[43] Bhattacharjee S Hamberger F Ravichandra A Miller M Nair A Affo S. Tumor restriction by type I collagen opposes tumor-promoting effects of cancer-associated fibroblasts. J Clin Invest. (2021) 131(11). doi: 10.1172/JCI146987 PMC8159701 33905375

[44] Chen Y Kim J Yang S Wang H Wu CJ Sugimoto H. Type I collagen deletion in αSMA(+) myofibroblasts augments immune suppression and accelerates progression of pancreatic cancer. Cancer Cell. (2021) 39(4):548–565.e6. doi: 10.1016/j.ccell.2021.02.007 33667385 PMC8423173

[45] Salimian N Peymani M Ghaedi K Hashemi M Rahimi E. Collagen 1A1 (COL1A1) and Collagen11A1(COL11A1) as diagnostic biomarkers in breast, colorectal and gastric cancers. Gene. (2024) 892:147867. doi: 10.1016/j.gene.2023.147867 37783295

[46] Karancsi Z Gregus B Krenács T Cserni G Nagy Á Szőcs-Trinfa KF. Digital whole slide image analysis of elevated stromal content and extracellular matrix protein expression predicts adverse prognosis in triple-negative breast cancer. Int J Mol Sci. (2024) 25(17). doi: 10.3390/ijms25179445 PMC11394775 39273393

[47] Imanbayev NM Iztleuov YM Kamyshanskiy YK Zhumasheva AV. Diagnostic and prognostic significance of keloid-like collagen remodeling patterns in the extracellular matrix of colorectal cancer. Pathol Oncol Res. (2024) 30:1611789. doi: 10.3389/pore.2024.1611789 38903488 PMC11186984

[48] Li X Sun X Kan C Chen B Qu N Hou N. COL1A1: A novel oncogenic gene and therapeutic target in malignancies. Pathol Res Pract. (2022) 236:154013. doi: 10.1016/j.prp.2022.154013 35816922

[49] Shi R Zhang Z Zhu A Xiong X Zhang J Xu J. Targeting type I collagen for cancer treatment. Int J Cancer. (2022) 151(5):665–83. doi: 10.1002/ijc.33985 35225360

[50] Heichler C Schmied A Enderle K Scheibe K Murawska M Schmid B. Targeting STAT3 signaling in COL1+ fibroblasts controls colitis-associated cancer in mice. Cancers (Basel). (2022) 14(6). doi: 10.3390/cancers14061472 PMC8946800 35326623

[51] Baldari S Di Modugno F Nisticò P Toietta G. Strategies for efficient targeting of tumor collagen for cancer therapy. Cancers (Basel). (2022) 14(19). doi: 10.3390/cancers14194706 PMC9563908 36230627

[52] Wang TH Hsia SM Shieh TM. Lysyl oxidase and the tumor microenvironment. Int J Mol Sci. (2016) 18(1). doi: 10.3390/ijms18010062 PMC5297697 28036074

[53] Bansode S Bashtanova U Li R Clark J Müller KH Puszkarska A. Glycation changes molecular organization and charge distribution in type I collagen fibrils. Sci Rep. (2020) 10(1):3397. doi: 10.1038/s41598-020-60250-9 32099005 PMC7042214

[54] Park S Choi S Shimpi AA Estroff LA Fischbach C Paszek MJ. Collagen mineralization decreases NK cell-mediated cytotoxicity of breast cancer cells *via* increased glycocalyx thickness. Adv Mater. (2024) 36(43):e2311505. doi: 10.1002/adma.202311505 38279892 PMC11471288

[55] Shen XT Chen ZC Wang XY Wang XF Xie SZ Zheng X. Establishment of homotrimer collagen type I signature and its association with clinical manifestation and tertiary lymphoid structures formation in liver cancer. Heliyon. (2024) 10(11):e31320. doi: 10.1016/j.heliyon.2024.e31320 38841477 PMC11152946

[56] Esfahani SA Ma H Krishna S Shuvaev S Sabbagh M Deffler C. Collagen type I PET/MRI enables evaluation of treatment response in pancreatic cancer in pre-clinical and first-in-human translational studies. Theranostics. (2024) 14(15):5745–61. 10.7150/thno.100116 PMC11426233 39346545

[57] Donmez Cakil Y Akbulut Z Maras H Gokceoglu Kayali D Gulhan Aktas R. Collagen type I induces a balance in the expression of anti- and pro-apoptotic genes in hepatocellular carcinoma cells %. Middle East J Cancer. (2022) 13(1):89–98. doi: 10.30476/mejc.2021.85875.1312

[58] Hurkmans DP Jensen C Koolen SLW Aerts J Karsdal MA Mathijssen RHJ. Blood-based extracellular matrix biomarkers are correlated with clinical outcome after PD-1 inhibition in patients with metastatic melanoma. J Immunother Cancer. (2020) 8(2). 10.1136/jitc-2020-001193 PMC7583811 33093157

[59] Stewart DC Brisson BK Dekky B Berger AC Yen W Mauldin EA. Prognostic and therapeutic implications of tumor-restrictive type III collagen in the breast cancer microenvironment. NPJ Breast Cancer. (2024) 10(1):86. 39358397 10.1038/s41523-024-00690-y PMC11447064

[60] Zeng H Li H Wang L You S Liu S Dong X. Recombinant humanized type III collagen inhibits ovarian cancer and induces protective anti-tumor immunity by regulating autophagy through GSTP1. Mater Today Bio. (2024) 28:101220. 10.1016/j.mtbio.2024.101220 PMC11405829 39290464

[61] Di Martino JS Nobre AR Mondal C Taha I Farias EF Fertig EJ. A tumor-derived type III collagen-rich ECM niche regulates tumor cell dormancy. Nat Cancer. (2022) 3(1):90–107. 35121989 10.1038/s43018-021-00291-9 PMC8818089

[62] Zhang H Ding C Li Y Xing C Wang S Yu Z. Data mining-based study of collagen type III alpha 1 (COL3A1) prognostic value and immune exploration in pan-cancer. Bioengineered. (2021) 12(1):3634–46. doi: 10.1080/21655979.2021.1949838 PMC8806444 34252356

[63] Willumsen N Jensen C Green G Nissen NI Neely J Nelson DM. Fibrotic activity quantified in serum by measurements of type III collagen pro-peptides can be used for prognosis across different solid tumor types. Cell Mol Life Sci. (2022) 79(4):204. doi: 10.1007/s00018-022-04226-0 35332383 PMC8948122

[64] Pedersen RS Thorlacius-Ussing J Raimondo MG Langholm LL Schett G Ramming A. Fibroblast activation protein (FAP)-mediated cleavage of type III collagen reveals serum biomarker potential in non-small cell lung cancer and spondyloarthritis. Biomedicines. (2024) 12(3). doi: 10.3390/biomedicines12030545 PMC10968340 38540158

[65] Bonnesoeur S Morin-Grognet S Thoumire O Le Cerf D Boyer O Vannier JP. Hyaluronan-based hydrogels as versatile tumor-like models: Tunable ECM and stiffness with genipin-crosslinking. J BioMed Mater Res A. (2020) 108(5):1256–68. doi: 10.1002/jbm.a.36899 32056374

[66] Guszcz T Sankiewicz A Gałek L Chilinska-Kopko E Hermanowicz A Gorodkiewicz E. Application of surface plasmon resonance imaging biosensors for determination of fibronectin, laminin-5, and type IV collagen in plasma, urine, and tissue of renal cell carcinoma. Sensors (Basel). (2024) 24(19). doi: 10.3390/s24196371 PMC11478812 39409411

[67] Nanashima A Hiyoshi M Imamura N Hamada T Tsuchimochi Y Shimizu I. Clinical significances of several fibrotic markers for prognosis in hepatocellular carcinoma patients who underwent hepatectomy. Transl Cancer Res. (2024) 13(5):2332–45. doi: 10.21037/tcr-24-94 PMC11170533 38881924

[68] Vautrin-Glabik A Devy J Bour C Baud S Choulier L Hoarau A. Angiogenesis inhibition by a short 13 amino acid peptide sequence of tetrastatin, the α4(IV) NC1 domain of collagen IV. Front Cell Dev Biol. (2020) 8:775. doi: 10.3389/fcell.2020.00775 32850867 PMC7431705

[69] Wu Y Liu X Zhu Y Qiao Y Gao Y Chen J. Type IV collagen α5 chain promotes luminal breast cancer progression through c-Myc-driven glycolysis. J Mol Cell Biol. (2023) 14(10). doi: 10.1093/jmcb/mjac068 PMC10077331 36484686

[70] Jansson M Lindberg J Rask G Svensson J Billing O Nazemroaya A. Prognostic value of stromal type IV collagen expression in small invasive breast cancers. Front Mol Biosci. (2022) 9:904526. doi: 10.3389/fmolb.2022.904526 35693557 PMC9174894

[71] Brett E Sauter M Timmins É Azimzadeh O Rosemann M Merl-Pham J. Oncogenic linear collagen VI of invasive breast cancer is induced by CCL5. J Clin Med. (2020) 9(4). doi: 10.3390/jcm9040991 PMC7230614 32252260

[72] Ho CM Chang TH Yen TL Hong KJ Huang SH. Collagen type VI regulates the CDK4/6-p-Rb signaling pathway and promotes ovarian cancer invasiveness, stemness, and metastasis. Am J Cancer Res. (2021) 11(3):668–90. PMC7994167 33791147

[73] Piao XM Hwang B Jeong P Byun YJ Kang HW Seo SP. Collagen type VI-α1 and 2 repress the proliferation, migration and invasion of bladder cancer cells. Int J Oncol. (2021) 59(1). doi: 10.3892/ijo.2021.5217 33982770

[74] Lee OE Le TM Chong GO Cho JJ Park NJ. The mclust analysis of tumor budding unveils the role of the collagen family in cervical cancer progression. Life (Basel). (2024) 14(8). doi: 10.3390/life14081004 PMC11355860 39202746

[75] Liu Q Zhang X Song Y Si J Li Z Dong Q. Construction and analysis of a reliable five-gene prognostic signature for colon adenocarcinoma associated with the wild-type allelic state of the COL6A6 gene. Transl Cancer Res. (2024) 13(5):2475–96. doi: 10.21037/tcr-23-463 PMC11170513 38881933

[76] Liu Y Murazzi I Fuller AM Pan H Irizarry-Negron VM Devine A. Sarcoma cells secrete hypoxia-modified collagen VI to weaken the lung endothelial barrier and promote metastasis. Cancer Res. (2024) 84(7):977–93. doi: 10.1158/0008-5472.CAN-23-0910 PMC10984776 38335278

[77] Yamaguchi K Nakayama J Yamamoto T Semba K Shirota T Yamamoto Y. Collagen induction of immune cells in the mammary glands during pregnancy. Physiol Genomics. (2024) 56(2):128–35. doi: 10.1152/physiolgenomics.00098.2023 PMC11281812 37955336

[78] Peng J Liu X Mao Y Lv M Ma T Liu J. Upregulation of collagen type x alpha 1 promotes the progress of triple-negative breast cancer *via* wnt/β-catenin signaling. Mol Carcinog. (2024) 63(8):1588–98. doi: 10.1002/mc.23747 38780151

[79] Chapman KB Prendes MJ Sternberg H Kidd JL Funk WD Wagner J. COL10A1 expression is elevated in diverse solid tumor types and is associated with tumor vasculature. Future Oncol. (2012) 8(8):1031–40. doi: 10.2217/fon.12.79 22894674

[80] Huang H Li T Ye G Zhao L Zhang Z Mo D. High expression of COL10A1 is associated with poor prognosis in colorectal cancer. Onco Targets Ther. (2018) 11:1571–81. doi: 10.2147/OTT.S160196 PMC5865565 29593423

[81] Li T Huang H Shi G Zhao L Li T Zhang Z. TGF-β1-SOX9 axis-inducible COL10A1 promotes invasion and metastasis in gastric cancer *via* epithelial-to-mesenchymal transition. Cell Death Dis. (2018) 9(9):849. doi: 10.1038/s41419-018-0877-2 30154451 PMC6113209

[82] Shen N Zhu S Zhang Z Yong X. High expression of COL10A1 is an independent predictive poor prognostic biomarker and associated with immune infiltration in advanced gastric cancer microenvironment. J Oncol. (2022) 2022:1463316. doi: 10.1155/2022/1463316 36276283 PMC9584694

[83] Wang C Wang J Chen S Li K Wan S Yang L. COL10A1 as a prognostic biomarker in association with immune infiltration in prostate cancer. Curr Cancer Drug Targets. (2024) 24(3):340–53. doi: 10.2174/1568009623666230817101809 37592784

[84] Wen Z Sun J Luo J Fu Y Qiu Y Li Y. COL10A1-DDR2 axis promotes the progression of pancreatic cancer by regulating MEK/ERK signal transduction. Front Oncol. (2022) 12:1049345. doi: 10.3389/fonc.2022.1049345 36530986 PMC9750160

[85] Xu S Liu D Qin Z Liang Z Xie H Yi B. Experimental validation and pan-cancer analysis identified COL10A1 as a novel oncogene and potential therapeutic target in prostate cancer. Aging (Albany NY). (2023) 15(24):15134–60. doi: 10.18632/aging.205337 PMC10781495 38147021

[86] Brodsky AS Xiong J Yang D Schorl C Fenton MA Graves TA. Identification of stromal ColXα1 and tumor-infiltrating lymphocytes as putative predictive markers of neoadjuvant therapy in estrogen receptor-positive/HER2-positive breast cancer. BMC Cancer. (2016) 16:274. doi: 10.1186/s12885-016-2302-5 27090210 PMC4835834

[87] Zhao CL Singh K Brodsky AS Lu S Graves TA Fenton MA. Stromal ColXα1 expression correlates with tumor-infiltrating lymphocytes and predicts adjuvant therapy outcome in ER-positive/HER2-positive breast cancer. BMC Cancer. (2019) 19(1):1036. 31675929 10.1186/s12885-019-6134-y PMC6825361

[88] Kahlert UD Shi W Strecker M Scherpinski LA Wartmann T Dölling M. COL10A1 allows stratification of invasiveness of colon cancer and associates to extracellular matrix and immune cell enrichment in the tumor parenchyma. Front Oncol. (2022) 12:1007514. doi: 10.3389/fonc.2022.1007514 36267978 PMC9577326

[89] Wu J Ouyang P Huang R Cui Y Yang Z Xu W. METTL16 promotes stability of SYNPO2L mRNA and leading to cancer cell lung metastasis by secretion of COL10A1 and attract the cancer-associated fibroblasts. Int J Biol Sci. (2024) 20(11):4128–45. doi: 10.7150/ijbs.95375 PMC11379079 39247832

[90] Guo Q Zheng M Xu Y Wang N Zhao W. MiR-384 induces apoptosis and autophagy of non-small cell lung cancer cells through the negative regulation of collagen α-1(X) chain gene. Biosci Rep. (2019) 39(2). doi: 10.1042/BSR20181523 PMC6356039 30442874

[91] Suzuki K Matsui Y Higashimoto M Kawaguchi Y Seki S Motomura H. Myxoid liposarcoma-associated EWSR1-DDIT3 selectively represses osteoblastic and chondrocytic transcription in multipotent mesenchymal cells. PloS One. (2012) 7(5):e36682. doi: 10.1371/journal.pone.0036682 22570737 PMC3343026

[92] Arai K Uehara K and Nagai Y. Expression of type II and type XI collagens in canine mammary mixed tumors and demonstration of collagen production by tumor cells in collagen gel culture. Jpn J Cancer Res. (1989) 80(9):840–7. 10.1111/j.1349-7006.1989.tb01724.x PMC5917859 2480942

[93] Wu YH Huang YF Wu PY Chang TH Huang SC Chou CY. The downregulation of miR-509-3p expression by collagen type XI alpha 1-regulated hypermethylation facilitates cancer progression and chemoresistance *via* the DNA methyltransferase 1/Small ubiquitin-like modifier-3 axis in ovarian cancer cells. J Ovarian Res. (2023) 16(1):124. doi: 10.1186/s13048-023-01191-5 37386587 PMC10308652

[94] Liu Z Lai J Jiang H Ma C Huang H. Collagen XI alpha 1 chain, a potential therapeutic target for cancer. FASEB J. (2021) 35(6):e21603. doi: 10.1096/fj.202100054RR 33999448 PMC12315952

[95] Nissen NI Kehlet S Johansen AZ Chen IM Karsdal M Johansen JS. Noninvasive prognostic biomarker potential of quantifying the propeptides of type XI collagen alpha-1 chain (PRO-C11) in patients with pancreatic ductal adenocarcinoma. Int J Cancer. (2021) 149(1):228–38. doi: 10.1002/ijc.33551 33687786

[96] Zhu J Weng Y Wang F Zhao J. LINC00665/miRNAs axis-mediated collagen type XI alpha 1 correlates with immune infiltration and malignant phenotypes in lung adenocarcinoma. Open Med (Wars). (2022) 17(1):1259–74. doi: 10.1515/med-2022-0478 PMC9281593 35892083

[97] Zeltz C Khalil M Navab R Tsao MS. Collagen type XI inhibits lung cancer-associated fibroblast functions and restrains the integrin binding site availability on collagen type I matrix. Int J Mol Sci. (2022) 23(19). doi: 10.3390/ijms231911722 PMC9569509 36233024

[98] Kashiwagi R Funayama R Aoki S Matsui A Klein S Sato Y. Collagen XVII regulates tumor growth in pancreatic cancer through interaction with the tumor microenvironment. Cancer Sci. (2023) 114(11):4286–98. doi: 10.1111/cas.15952 PMC10637054 37688308

[99] Lothong M Sakares W Rojsitthisak P Tanikawa C Matsuda K Yodsurang V. Collagen XVII inhibits breast cancer cell proliferation and growth through deactivation of the AKT/mTOR signaling pathway. PloS One. (2021) 16(7):e0255179. doi: 10.1371/journal.pone.0255179 34293053 PMC8297889

[100] Crespo-Bravo M Thorlacius-Ussing J Nissen NI Pedersen RS Boisen MK Liljefors M. Levels of type XVII collagen (BP180) ectodomain are elevated in circulation from patients with multiple cancer types and is prognostic for patients with metastatic colorectal cancer. BMC Cancer. (2023) 23(1):949. doi: 10.1186/s12885-023-11470-5 37803411 PMC10557271

[101] Tuusa J Kokkonen N and Tasanen K. BP180/Collagen XVII: A molecular view. Int J Mol Sci. (2021) 22(22). doi: 10.3390/ijms222212233 PMC8623354 34830116

[102] Meijer RPJ Neijenhuis LKA Zeilstra AP Roerink SF Bhairosingh SS Hilling DE. Data-driven identification of targets for fluorescence-guided surgery in non-small cell lung cancer. Mol Imaging Biol. (2023) 25(1):228–39. doi: 10.1007/s11307-022-01791-5 36575340

[103] Mak KM Png CY Lee DJ. Type V collagen in health, disease, and fibrosis. Anat Rec (Hoboken). (2016) 299(5):613–29. doi: 10.1002/ar.23330 26910848

[104] Zhu G Wang Y Wang Y Huang H Li B Chen P. Myofibroblasts derived type V collagen promoting tissue mechanical stress and facilitating metastasis and therapy resistance of lung adenocarcinoma cells. Cell Death Dis. (2024) 15(7):493. doi: 10.1038/s41419-024-06873-6 38987529 PMC11237033

[105] Chen X Ma C Li Y Liang Y Chen T Han D. COL5A1 promotes triple-negative breast cancer progression by activating tumor cell-macrophage crosstalk. Oncogene. (2024) 43(23):1742–56. doi: 10.1038/s41388-024-03030-3 38609499

[106] Tan Y Chen Q Xing Y Zhang C Pan S An W. High expression of COL5A2, a member of COL5 family, indicates the poor survival and facilitates cell migration in gastric cancer. Biosci Rep. (2021) 41(4). doi: 10.1042/BSR20204293 PMC8039095 33739392

[107] Wang J Jiang YH Yang PY Liu F. Increased collagen type V α2 (COL5A2) in colorectal cancer is associated with poor prognosis and tumor progression. Onco Targets Ther. (2021) 14:2991–3002. doi: 10.2147/OTT.S288422 33981148 PMC8107053

[108] Nissen NI Johansen AZ Chen IM Jensen C Madsen EA Hansen CP. High serum levels of the C-propetide of type V collagen (PRO-C5) are prognostic for short overall survival in patients with pancreatic ductal adenocarcinoma. Front Mol Biosci. (2023) 10:1158058. doi: 10.3389/fmolb.2023.1158058 36968276 PMC10036831

[109] Duan X Ye D Yuan J Guan B Guan W Liu S. COL5A1 overexpression correlates with poor prognosis in human cervical cancer. Int J Biol Markers. (2024) 39(3):265–73. doi: 10.1177/03936155241265976 39043220

[110] Gong H Wu W Fang C He D. CircBFAR correlates with poor prognosis and promotes laryngeal squamous cell cancer progression through miR-31-5p/COL5A1 axis. Laryngoscope Investig Otolaryngol. (2022) 7(6):1951–62. doi: 10.1002/lio2.966 PMC9764770 36544920

[111] Poomsawat S Kariya A Nimmanon T Kosanwat T Juengsomjit R Sirima S. Diagnostic potential of type VII collagen during oral carcinogenesis. J Appl Oral Sci. (2023) 31:e20220486. doi: 10.1590/1678-7757-2022-0486 37194793 PMC10219628

[112] Oh SE Oh MY An JY Lee JH Sohn TS Bae JM. Prognostic value of highly expressed type VII collagen (COL7A1) in patients with gastric cancer. Pathol Oncol Res. (2021) 27:1609860. doi: 10.3389/pore.2021.1609860 34512204 PMC8426344

[113] Port H He Y Karsdal MA Madsen EA Bay-Jensen AC Willumsen N. Type IX collagen turnover is altered in patients with solid tumors. Cancers (Basel). (2024) 16(11). doi: 10.3390/cancers16112035 PMC11171364 38893155

[114] Crespo-Bravo M Hettich A Thorlacius-Ussing J Cox TR Karsdal MA Willumsen N. Type XII collagen is elevated in serum from patients with solid tumors: a non-invasive biomarker of activated fibroblasts. Clin Exp Med. (2024) 24(1):166. doi: 10.1007/s10238-024-01431-y 39048763 PMC11269340

[115] Papanicolaou M Parker AL Yam M Filipe EC Wu SZ Chitty JL. Temporal profiling of the breast tumour microenvironment reveals collagen XII as a driver of metastasis. Nat Commun. (2022) 13(1):4587. doi: 10.1038/s41467-022-32255-7 35933466 PMC9357007

[116] Wang C Zhu J Ma J Meng X Ma Z Fan F. Optical coherence elastography and its applications for the biomechanical characterization of tissues. J Biophotonics. (2023) 16(12):e202300292. doi: 10.1002/jbio.202300292 37774137

[117] Zhang C Lin F Zhang Y Yang H Lin D He J. Super-resolution second-harmonic generation imaging with multifocal structured illumination microscopy. Nano Lett. (2023) 23(17):7975–82. doi: 10.1021/acs.nanolett.3c01903 37642385

[118] Thorlacius-Ussing J Jensen C Nissen NI Cox TR Kalluri R Karsdal M. The collagen landscape in cancer: profiling collagens in tumors and in circulation reveals novel markers of cancer-associated fibroblast subtypes. J Pathol. (2024) 262(1):22–36. doi: 10.1002/path.6207 37728068

